# The UFM1 Conjugation System: A Master Regulator of Cellular Stress Surveillance in Human Disease

**DOI:** 10.3390/biology15050382

**Published:** 2026-02-26

**Authors:** Meiqian Kuang, Haigang Xu, Hongjun Huang, Caifang Ren, Pan Huang, Aihua Gong

**Affiliations:** 1 School of Medicine, Jiangsu University, Zhenjiang 212013, China; 2212413120@stmail.ujs.edu.cn (H.X.); rcf@ujs.edu.cn (C.R.); phuang@ujs.edu.cn (P.H.); 2 The First Clinical Medical College, Hainan Medical University, Haikou 570100, China; 18789254812@163.com

**Keywords:** UFM1, UFM1 conjugation system, UFMylation, stress response, diseases, cancer

## Abstract

Post-translational modification (PTM) encompasses diverse modifications, including phosphorylation, methylation, ubiquitin-like modifications (UBLs), and so on, which profoundly influence cellular functions. UFMylation is a recently identified ubiquitin-like modification, which is mediated by the Ubiquitin-like Ubiquitin Fold Modifier 1 (UFM1) conjugation system. The UFM1 conjugation system comprises UFM1, Ubiquitin-like protein activating enzyme 5 (UBA5), UFM1-conjugating enzyme 1 (UFC1), UFM1-specific ligase 1 (UFL1), UFM1-specific protease 1 (UFSP1), UFM1-specific protease 2 (UFSP2), UFM1-binding protein 1 (UFBP1), and CDK5 regulatory subunit-associated protein 3 (CDK5RAP3). Accumulating research has demonstrated that the UFM1 conjugation system regulates various cellular stress responses, including endoplasmic reticulum (ER) stress, protein trafficking, DNA damage repair, and autophagy. Additionally, abnormal stress adaptations of the UFM1 conjugation system contribute to the pathophysiological complications of inflammatory diseases and cancer, underscoring its significance as a key regulatory node in human health and disease. Therefore, this review provides a comprehensive exploration of the structural characteristics of UFM1 conjugation system members and the mechanistic roles of UFMylation by UFM1 conjugation system-mediated diseases related to cellular stress responses, which will not only facilitate the identification of novel diagnostic and prognostic indicators but also enable the identification of specific therapeutic targets for UFM1 conjugation system-related diseases.

## Simple Summary

The cellular stress response plays a critical role in maintaining cell homeostasis when cells are exposed to various internal and external stressors that threaten their survival. UFMylation, mediated by the UFM1 conjugation system, a newly identified ubiquitin-like modification pathway, is essential for coordinating these stress responses, including endoplasmic reticulum stress, DNA damage repair, and autophagy, etc. Dysregulation of the UFM1 conjugation system disrupts substrate protein stability and stress signaling fidelity, contributing to the development of diverse human diseases. Our review summarizes recent, newly found evidence of how UFM1 conjugation system-mediated UFMylation governs cellular stress response surveillance and highlights its potential as a promising therapeutic target in various stress-associated inflammation disorders and cancers.

## 1. Introduction

Post-translational modification (PTM) is pivotal regulators of cellular function, encompassing diverse mechanisms such as phosphorylation, methylation, and ubiquitin-like modifications (UBLs) [1]. Among these, UFMylation is a newly identified UBL pathway mediated by the Ubiquitin Fold Modifier 1 (UFM1) conjugation system, which has emerged as a critical player in cellular stress adaptation [2]. Masaaki Komatsu et al. initially reported that UFM1 was discovered by experimental investigations utilizing HEK293 cells and murine tissues in 2004, and it plays an important role in various stress responses in mediated UFMylation, thereby maintaining stress response under pathological perturbations [3]. Since the discovery of UFM1 in 2004, a number of UFM1 conjugation system members have been identified, including E1-activating enzyme Ubiquitin-like protein-activating enzyme 5 (UBA5), the conjugating enzyme (E2) UFM1-conjugating enzyme 1 (UFC1), and the ligase (E3) UFM1-specific ligase 1 (UFL1). In addition, two dedicated proteases, UFM1-specific protease 1 (UFSP1) and UFM1-specific protease 2 (UFSP2), were identified to reverse this modification. Key regulatory and effector proteins within the system, such as UFM1-binding protein 1 (UFBP1), and CDK5 regulatory subunit-associated protein 3 (CDK5RAP3), have also been characterized [3,4,5,6,7,8]. In addition to its core components, the UFM1 conjugation system encompasses a variety of cofactors, including Activating signal cointegrator 1 (ASC1), Recombinant human ribosomal protein L26 (RPL26), and Histone 4 (H4), among others, which expand the functional scope of UFMylation across diverse cellular compartments [9,10,11]. These molecular players collectively orchestrate dynamic UFMylation processes that fine-tune stress signaling networks across multiple diseases.

**Figure 1:**
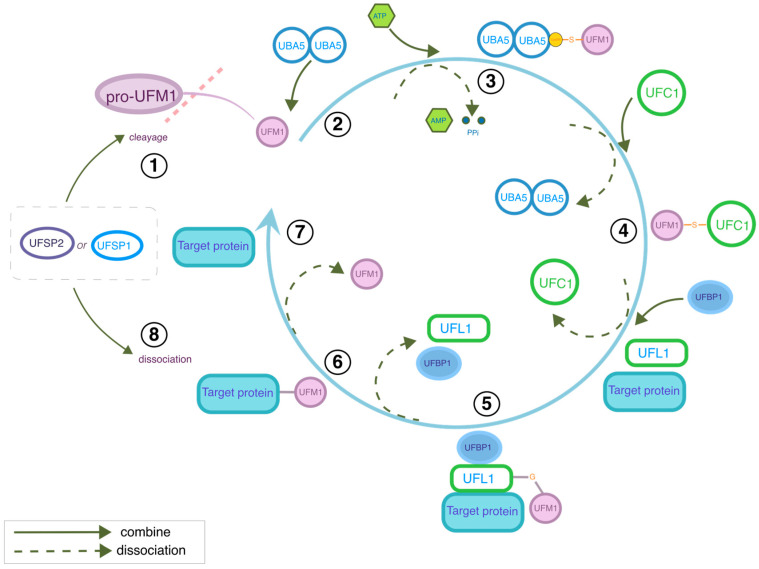
Schematic diagram of UFMylation mediated by UMF1 conjugation system. Pro-UFM1 hydrolysis is mediated by UFSP1 and UFSP2 to generate mature UFM1. Subsequently, UBA5 undergoes ATP-dependent structural reorganization within its adenylation domain, positioning the catalytic Cys250 and enabling a nucleophilic attack by Cys250 on UFM1 to form a UBA5-S-UFM1 thioester bond. Following successful activation of UFM1, UFC1 binds to the Cys116 residue on one UBA5 subunit and accepts the UFM1 moiety from the other UBA5 subunit. UFL1 then interacts with UFC1 and UFBP1 through its N-terminal domain to form a ternary complex, ultimately transferring UFM1 to target proteins to complete UFMylation modification. After modification, UFM1 is recycled through a UFSP1/UFSP2-mediated decoupling process.

Similarly to ubiquitination, the PTM process known as UFMylation involves ATP-dependent activation and conjugation of UFM1 to its specific target proteins through a tightly regulated enzymatic cascade [12,13]. UFM1 activation requires proteolytic processing of its precursor form (Pro-UFM1). The C-terminal Gly-Ser-Cys extension of Pro-UFM1 is proteolytically cleaved by UFSP1 and UFSP2, thereby exposing the terminal glycine residue essential for subsequent conjugation [6,14]. Activated UFM1 is then adenylated by the E1 enzyme UBA5, which undergoes ATP-dependent conformational rearrangement within its adenylation domain. The reoriented catalytic cysteine residue (Cys250) performs a nucleophilic attack on the UFM1-AMP intermediate, forming a transient thioester bond between UBA5 and mature UFM1 [15,16,17]. The activated UFM1 is then transferred to the UFC1 via trans-thioesterification [18]. Thereafter, the E3 ligase complex, composed of UFL1 and its cofactor UFBP1, facilitates the conjugation of UFM1 to lysine residues on substrate proteins through coordinated E2 and E3 interactions [18,19,20]. This modification cycle is dynamically regulated by UFSP1/UFSP2-mediated deconjugation, which hydrolyzes UFM1-substrate linkages to recycle free UFM1 for subsequent rounds of modification (see Figure 1) [6,14]. Such enzymatic precision ensures spatiotemporal control of UFMylation, enabling its involvement in diverse cellular processes while preserving the overall homeostasis of the UFM1 conjugation system.

In recent years, growing evidence has suggested that the UFM1 conjugation system exerts a significant influence on multiple cellular stress responses triggered by intrinsic and extrinsic stimuli, including endoplasmic reticulum (ER) stress, ER-phagy, oxidative stress, protein quality control, apoptosis, and DNA damage [21,22,23,24,25]. Dysregulation of cellular stress response homeostasis represents a shared pathogenic mechanism underlying various human disorders. Consistently, emerging data has demonstrated that the function of UFM1 conjugation system members is relevant to a wide spectrum of human disorders, including tumors and inflammatory disorders in various systems, such as the circulatory, neurological, digestive, reproductive, urinary, and skeletal systems [26,27]. However, regulatory mechanisms of the UFM1 conjugation system and substrate diversity remain to be thoroughly elucidated. Therefore, this review systematically presents a comprehensive overview of the biological functions of UFM1-mediated UFMylation and its regulatory role in cellular stress adaptation across disease states while outlining critical directions for future mechanistic and translational investigations.

## 2. Structural Characteristics of the UFM1 Conjugation System

### 2.1. UFM1

UFM1 is a ubiquitin-like protein modifier that displays remarkable structural conservation across animals and plants. It comprises 85 amino acid residues with a calculated molecular mass of 9.9 kDa [28]. Once UFM1 is activated, the UFM1 conjugation system regulates diverse cellular processes and disease pathways through the modification of specific substrate proteins [29]. Although UFM1 shares only ~18% amino acid sequence identity with ubiquitin (UB), both adopt the characteristic β-grasp fold, a hallmark of ubiquitin-like modifiers [2,30]. UFM1 is presumed to adopt a ubiquitin-like fold, in which the b-strands are composed of Lys3-Thr9 (designated as β1), Val20-Pro24 (β2), Ser47-Thr51 (β3), and Glu73-Pro78 (β5) in humans [22,31,32]. Meanwhile, the a-helices are formed by Pro29-Glu39 (α1) and Ala63-Lys69 (α2) [22,31]. In contrast to the canonical ubiquitin fold, theβ4-strand that typically connects β3 and α2 is poorly defined in UFM1. This structural ambiguity is attributed to the presence of two glycine residues (Gly54 and Gly56), which destabilize β-sheet formation in this region [22,31,33]. Notably, UFM1 lacks the conserved cluster of acidic residues on the α1 surface that is typical of ubiquitin and NEDD8, a feature known to facilitate E2 and E3 enzyme binding [22,33]. It is suggested that UFM1 demonstrates distinct substrate selectivity across species, reflecting functional divergence in its post-translational regulatory roles [34,35]. This multi-layered quality control paradigm underscores the fact that UFM1’s role provides novel insights into post-translational regulation of proteostasis, with broad implications for understanding pathological dysregulation in cancer and stress-related disorders [22,36,37,38,39].

### 2.2. UBA5

UBA5, alternatively designated UBE1DC1, refers to the full-length protein comprising 404 amino acids with a predicted molecular weight of 46.2 kDa [40]. As a non-canonical E1 enzyme, UBA5 differs from classical E1 enzymes in that classical E1 enzymes harbor both the first and second catalytic cysteine subdomains (FCCH and SCCH), as well as a ubiquitin-fold domain (UFD), for E2 recruitment. Instead, UBA5 retains a single adenylation domain with reduced ATP-binding capacity that must dimerize to achieve high-affinity ATP binding [15,41,42]. The N-terminal extension of the long UBA5 isoform directly contributes to ATP binding and modulates adenylation activity, reflecting a divergent catalytic mechanism relative to canonical E1 enzymes [15,40,42]. Because UBA5 lacks the UFD, its C-terminal region instead mediates UFM1 transfer to UFC1, illustrating a distinct noncanonical activation strategy [12,18,43]. Interestingly, UBA5 can exhibit a “reverse retrotransfer” reaction in which, under overexpression conditions of UBA5, UFM1 is transferred back from E2 (UFC1) to UBA5. This phenomenon is potentially associated with the flexible architecture of UBA5’s active site or the absence of structural constraints that restrict such reverse transfer in classical E1 enzymes [15,44]. Furthermore, the UBA5-UFC1 complex formation diverges from classical E1-E2 interactions, possibly relying on unique helix–helix interfaces rather than conserved hydrophobic pockets or charge complementarity [15,36,37]. These structural and functional idiosyncrasies underscore the evolutionary specialization of UBA5 as a dedicated E1 enzyme uniquely adapted to the UFMylation pathway.

### 2.3. UFC1

UFC1, alternatively termed HSPC155, is a 167-amino-acid protein with an estimated molecular weight of 19.4 kDa [3,45]. Structural analyses demonstrated that UFC1 shares only ~15% sequence identity with canonical E2 enzymes, and UFC1 adopts a conserved E2-like fold comprising a central core domain containing a four-stranded antiparallel β-sheet surrounded by five strategically positioned α-helices [15]. UFC1 contains a highly conserved catalytic cysteine (putatively mapped to Cys116) that localizes within a solvent-exposed flexible loop, and this cysteine mediates the transfer of UFM1 thioester bonds [16,46]. Structural and biochemical studies further indicate that UFC1’s unique N-terminal extension consists of an α1-helix (residues 1–11) and an adjacent loop region (residues 12–24). Within this loop region, Val9/Val10 form hydrophobic interactions with core domain residues His136/Ala139, but they remain functionally inactive in mediating E1-E2 complex assembly or catalytic thioesterification [43,47,48,49]. This hydrophobic interaction is critical for UFC1’s thermostability but is dispensable for its catalytic activity during E1-E2 complex assembly [44,46]. Consistent with this notion, clinical studies on mutations showed that Arg55Gln mutation in this region disrupts UFC1’s tertiary structure, leading to extensive UFMylation defects and neurodevelopmental disorders [50,51]. Biophysical studies indicated that the conformational dynamics of UFC1 regulate its competitive binding to UBA5 and UFL1, and changes in UFC1’s charge state modify its binding preferences [43]. This remarkable evolutionary plasticity demonstrates how tertiary structural conservation can be maintained despite limited sequence homology [47,48,49]. This functional decoupling identifies the α1-helix as a structural stabilizer distinct from conserved enzymatic machinery. While the functional significance of this unique structural feature remains incompletely resolved, it may be involved in modulating conformational stability and intermolecular interactions [18,19].

### 2.4. UFL1

UFL1, also termed RCAD, KIAA0776, NLBP, or Maxer, consists of 794 amino acids with an approximate molecular weight of 90 kDa [4]. Unlike classical E3 ligases, which typically harbor a Really Interesting New Gene (RING), Homologous to E6AP C-Terminus (HECT), or RING-between-RING domains to catalyze ubiquitin or ubiquitin-like transfer, UFL1 lacks all canonical E3 catalytic motifs, defining it as a non-canonical, scaffold-type E3 ligase within the UFMylation [52,53,54,55,56]. Rather than directly catalyzing UFM1 transfer, UFL1 must form a complex with the adaptor protein UFBP1 to activate the E2 enzyme UFC1 through conformational remodeling [18,40]. This UFL1/UFBP1 complex enables UFM1 transfer via a mechanism distinct from canonical E3 ligase catalytic modes, as it lacks the characteristic catalytic cysteine residues in its conserved N-terminal domain [18,43,57]. Critical hydrophobic residues within this domain mediate UFC1 binding, while its non-catalytic nature is further evidenced by structural comparisons to traditional E3 ligases [18,57]. The N-terminal region of UFL1 contains hydrophobic residues that mediate UFC1 engagement, while its C-terminal portion contributes to substrate selectivity through cooperative interactions with UFBP1 [18,58]. UFL1 contains multiple tandem PCI-like winged helix (WH) domains forming a rigid scaffold, stabilized by complementary interactions between adjacent WH domains (pWH-pWH’ complex) [18,58]. This architecture promotes activation of UFC1 and enables the transfer of UFM1 to substrates [18,40,59]. While the minimal catalytic domain of UFL1 is sufficient for UFC1 activation, additional WH domains likely refine substrate specificity. Functionally, the UFL1–UFBP1 complex acts as a non-catalytic scaffold that promotes UFM1 transfer to substrate proteins via spatial proximity, reminiscent of small ubiquitin-related modifier E3 ligases, but evolutionarily distinct owing to its WH domain organization [10,11,48]. This innovative structural design not only broadens our understanding of E3 ligase diversity but also provides new insights into the regulatory mechanisms of UFMylation and its implications in neurodevelopmental disorders and disease-related mutations.

### 2.5. UFSP1 and UFSP2

UFSP1 and UFSP2 are cysteine proteases that control both the activation and deconjugation of UFMylation, thereby ensuring dynamic control of the UFM1 conjugation system. Despite sharing catalytic core features, UFSP1 and UFSP2 display marked differences in molecular weight, domain architecture, and subcellular localization, which underlie their functional specialization. UFSP1 is a relatively small protein, predominantly cytosolic protease, whereas UFSP2 is a considerably larger protein that harbors an extended N-terminal region absent in UFSP1, a structural feature that underlies their functional divergence [60]. UFSP1 and UFSP2 both adopt a papain-like fold and harbor an evolutionarily conserved Cys-His-Asp catalytic triad, which mediates proteolytic cleavage of the C-terminal extension of pro-UFM1 to expose the terminal glycine required for conjugation [6,60,61]. While UFSP1 and UFSP2 catalyze Pro-UFM1 maturation and deconjugate UFM1 from substrates, their functional divergence stems from structural and localization differences [6,60]. Structural analyses further revealed that UFSP2 has an additional 136-amino acid N-terminal domain that is absent in UFSP1 [60]. The MPN fold in this domain exhibits a seven-stranded β-sheet flanked by α5 and α8 helices oriented perpendicularly, which enables ER membrane localization and substrate specificity [21,62]. Evidence suggests that the extended N-terminal region of UFSP2 coordinates with the tail-anchored cofactor ODR4 to ensure proper subcellular positioning [10,63]. This ER-specific positioning enables UFSP2 to preferentially deconjugate UFM1 from ribosomal targets like RPL26, thereby modulating ER-phagy and quality control during ribosomal stalling [21]. UFSP2 is responsible for removing UFM1 from conjugated substrates such as UFBP1 and ribosomal protein RPL26 [6,60,61]. In contrast, UFSP1 primarily functions in the cytosol and plays a key role in maintaining UFMylation flux by removing inhibitory auto-UFMylation from the E2 enzyme UFC1, thereby promoting pathway activation [6]. This functional partitioning allows UFSP1 to regulate global UFMylation capacity, while UFSP2 fine-tunes substrate-specific deUFMylation within the ER compartment. Different structural and localization-based characteristics between UFSP1 and UFSP2 establishes a compartmentalized regulatory mechanism, in which UFM1 precursor maturation, substrate deconjugation, and pathway recycling are spatially segregated. These molecular mechanisms enable precise spatiotemporal control of UFMylation dynamics, ensuring balanced proteostasis [6,62,64].

### 2.6. UFBP1

UFBP1, also termed DDRGK1, C20orf11676, or Dashurin, is a 35.6 kDa modular protein comprising 314 amino acids [65]. UFBP1 contains an N-terminal signal peptide (13–26 residues) that guides ER localization through structurally conserved features, even though this region shows limited sequence conservation across species [19,20,66]. The C-terminal region contains a PCI domain, which facilitates protein–protein interactions necessary for coordinating UFMylation dynamics [7,18,19]. Within this domain, Lys267 (Lys268 in humans) serves as the primary site for UFM1 conjugation [67]. Together, these structural elements enable UFBP1 to spatially integrate ER membrane targeting with enzymatic activity, ensuring precise regulation of substrate modification and cellular homeostasis [19].

### 2.7. CDK5RAP3

CDK5RAP3 (also referred to as C53/LZAP) is a 53 kDa multidomain protein that adopts a distinctive dumbbell-like architecture driven by its α-helical composition [18]. The central structure of CDK5RAP3 is two elongated antiparallel α-helices forming a coiled-coil core, flanked by globular domains at both termini [18,19]. There are three strategically positioned regions in CDK5RAP3 that maintain functional specificity: (1) a C-terminal UFM1/UFBP1-binding domain (UUBD) that facilitates the association with the ubiquitin-like protein UFM1 and its adaptor protein UFBP1; (2) a C-terminal ribosomal association domain (RBD) that interacts with RPL26; and (3) an additional interface mediating UFL1/UFBP1 complex engagement [18,20,68,69].This architecture supports multiple interaction interfaces that enable CDK5RAP3 to function as an integrative adaptor within the UFMylation mediated by the UMF1 conjugation system. During ER stress, UUBD enables the formation of a ternary CDK5RAP3, UFL1, and UFBP1 complex by engaging the UFM1-UFBP1 conjugate [19,20,38]. This complex acts as a sensor of ribosomal stalling, triggering the activation of ER-associated quality control pathways to promote the degradation of aberrant proteins [9,19]. During episodes of ribosomal collisions or translational arrest at the ER membrane, the RBD selectively recognizes RPL26 that has undergone UFMylation, thereby linking ribosome-associated quality control to ER-localized UFMylation. [19,20]. Notably, this interplay further regulates RPL26 UFMylation status, thereby influencing ribosome-associated quality control (RQC) mechanisms and cellular tolerance to ER stress [9,19,38]. The ternary complex not only facilitates but also spatially coordinates these functional domains, ensuring precise enzymatic activity and substrate recognition [19,20,38]. Such integrative structural organization underscores CDK5RAP3’s essentiality in maintaining UFMylation fidelity, bridging ribosomal function with ubiquitin-like protein signaling through its mechanically coupled domains [18,19]. The details of members of the UFM1 conjugation system are represented in Table 1.

## 3. Biological Role of UFM1 Conjugation System in Various Stress Responses

### 3.1. The UFM1 Conjugation System Exerts Bidirectional Control over ER Stress Responses

The UFM1 conjugation system has emerged as a pivotal regulator of ER stress signaling, exerting direct control over the activation and resolution of the unfolded protein response (UPR). Rather than functioning as a passive post-translational modification, UFMylation dynamically modulates multiple UPR branches, thereby governing whether ER stress culminates in adaptive recovery or apoptotic elimination [71,121,122]. ER stress that exceeds cellular adaptive capacity prompts the UFM1 conjugation system to initiate apoptotic pathways, thereby eliminating irreparably damaged cells and maintaining organismal integrity [36,37]. Notably, chronic ER stress induces UFM1 conjugation system dysfunction, creating a self-amplifying cycle that perpetuates ER stress through the large accumulation of unfolded proteins [123,124]. Upon sensing ER stress, the UFM1 conjugation system modulates UPR activation through three principal pathways, including the ATF6 pathway, the IRE1-XBP1 pathway, and the PERK-eIF2α-ATF4 pathway [19,45,125].

ATF6 is an ER-resident transmembrane transcription factor; although current studies have not yet identified the exact lysine residue(s) on ATF6 that undergo UFMylation, several mechanistic clues suggest how the UFM1 conjugation system may influence ATF6 activation. Recent studies have demonstrated that Bombyx mori UFBP1 (BmUFBP1) promotes viral proliferation by UFMylation-mediated modulation of BmATF6 activation and intracellular trafficking [65,126,127,128]. Knockdown of BmUFBP1 attenuates ATF6 signaling and downregulates ER chaperones, whereas application of an ATF6-specific agonist restores BmBIP and BmGRP94 expression, confirming its regulatory role. Conversely, BmUFBP1 overexpression enhances ATF6 pathway activity, alleviates ER stress, suppresses apoptosis, and thereby promotes viral replication [127,129]. Co-immunoprecipitation assays confirmed a direct physical interaction between BmUFBP1 and BmATF6, with UFMylation governing either proteolytic cleavage of ATF6’s during ER-Golgi translocation or its transcriptional activity to fine-tune the intensity of the UPR [65,128,130]. These findings highlight that the UFM1 conjugation system modulates the activation and trafficking of ATF6 pathway through adapters such as UFBP1. Importantly, while the UFMylation sites on ATF6 remain unidentified, structural and biochemical studies in mammalian systems show that UFBP1 contains a conserved UFM1-binding region and functions as a scaffold that recruits UFL1 to ER-associated protein complexes [65,128,130]. This scaffolding role provides a mechanistic basis for how UFMylation machinery may influence ATF6 activation indirectly, by stabilizing ER homeostasis.

The IRE1-XBP1 pathway represents another major target of UFM1-mediated regulation [131]. Evidence indicated that the UFM1 conjugation system stabilizes IRE1α through covalent UFMylation of UFBP1, which interacts with the transmembrane helix bundle residues 450–465 of IRE1α, thereby establishing a structural basis for UFM1-mediated regulation of ER stress signaling [132,133]. UFM1-mediated UFMylation generates a steric stabilization interface between UFBP1 and IRE1α, thereby preventing ER stress-induced degradation of IRE1α [133].This stabilization preserves IRE1α enzymatic activity, ensuring proper XBP1 mRNA splicing and continuous production of the transcriptionally active XBP1s isoform [133,134]. XBP1s subsequently drives the expression of ER chaperones, including GRP78, Protein Disulfide Isomerase (PDI), and folding catalysts, thereby enhancing ER’s proteostatic capacity [135,136,137]. Crucially, UFBP1 ablation reduces IRE1α protein levels and impairs UFM1-mediated protection, exacerbating ER stress [133,134]. Notably pathological implications emerge when the UFM1 conjugation system components are compromised.

Under ER stress conditions, PERK phosphorylates eIF2α, which transiently attenuates global protein synthesis while selectively enabling ATF4 translation [138,139]. ATF4 subsequently upregulates genes involved in redox homeostasis, amino acid metabolism, and ER stress adaptation, thereby initiating cytoprotective programs. Paradoxically, loss of UFM1 disrupts PERK regulatory checkpoints, triggering PERK hyperactivation through uncontrolled oligomerization and autophosphorylation [55,140,141]. This results in persistent phosphorylation of eIF2α, which exacerbates translational suppression while promoting ATF4-driven expression of C/EBP Homologous Protein (CHOP). Consequently, the pathway’s output shifts from protective to pro-apoptotic [141]. In summary, accumulating studies have demonstrated that the UFM1 conjugation system regulates the ER stress response though the ATF6 pathway, the IRE1-XBP1 pathway, and the PERK-eIF2α-ATF4 pathway, yet it exhibits significant bidirectionality in its functions. It not only aids cells in restoring ER homeostasis but also triggers cellular apoptosis when necessary. However, a critical scientific question remains unresolved: how does the UFM1 conjugation system precisely distinguish between “adaptable” and “apoptosis-requiring” ER stress intensities, and further achieve the pathway switch from “adaptation” to “apoptosis”?

### 3.2. UFM1 Conjugation System-Mediated UFMylation Regulates Nascent Polypeptide Chains and Mature Proteins in Protein Quality Control

Proteins are essential molecules for sustaining life processes, and all cells possess a Protein Quality Control (PQC) system to monitor proper protein assembly and selectively degrade misfolded polypeptides [142]. PQC is mediated by multiple mechanisms. Endoplasmic reticulum-associated degradation (ERAD) primarily surveys mature proteins, while RQC targets nascent polypeptide chains on the ribosome [143,144,145]. Notably, both systems rely on the UFM1 pathway to execute their protein quality control roles. HMG-CoA reductase degradation protein 1 (HRD1) is a ubiquitin ligase localized to the ER and serves as a core component of the ERAD machinery [146]. HRD1 catalyzes ubiquitin conjugation to misfolded or unfolded proteins, marking them for proteasomal degradation [147,148,149]. Upon exposure to protein misfolding stress, HRD1 is covalently modified by UFM1 at lysine 610 (K610), a cytosolic residue whose spatial positioning enables UFMylation [150]. This PTM represents a critical regulatory step for HRD1 functionality, triggering the formation of transmembrane channels [146,150,151,152]. UFMylation enhances HRD1’s ubiquitination activity, then HRD1 post-translationally modified by UFMylation, and then UFMylation of HRD1 activated eIF2α, accelerating clearance of misfolded proteins and preserving ER homeostasis [20,150,153]. However, conditions such as UFL1 deficiency or pathogenic K610R mutations in HRD1 abolish HRD1 UFMylation [150]. This impairment disrupts its protein homeostasis function in degrading client substrates including T cell receptor alpha chain and Monoacylglycerol acyltransferase-2, resulting in pathological ER retention of misfolded proteins [154,155]. Persistent protein accumulation activates the IRE1α-XBP1 and PERK signaling pathways, culminating in chronic ER stress that triggers Caspase-3/8 activation and apoptosis [150]. This mechanistic framework implicates UFMylation of HRD1 dysregulation in the pathogenesis of metabolic disorders, cancer, and neurodegenerative diseases.

The ERAD system primarily monitors mature proteins or polypeptides, while surveillance of those bound to the ER membrane relies on the RQC pathway mediated by RPL26 [156,157]. RPL26, a component of the 60S large ribosomal subunit, localizes near the polypeptide exit tunnel and normally participates in ribosome assembly and translation, facilitating nascent chain elongation and translocation [9,20]. When ribosomal translation stalls due to mRNA damage, amino acid deprivation, or other defects, RPL26 acts as a critical checkpoint to coordinate clearance of aberrant nascent chains [9,20,145]. During translation stalling, the UFM1-regulatory E3 ligase complex, composed of UFL1, UFBP1, and CDK5RAP3, initiates a “writing module” phase, functioning as an E3 ligase that wraps around the 60S subunit via its C-shaped clamp-like structure to conjugate UFM1 to RPL26. Following UFMylation of RPL26, the UFM1-regulatory E3 ligase complex undergoes conformational changes, transitioning to a “reading module” phase [9,20,158]. Here, the UFM1 recognition module (potentially involving UFBP1 or CDK5RAP3) specifically binds the modified RPL26, promoting dissociation of the UFMylated 60S subunit from the Sec61 complex (SEC61) translocon [20,145]. Aberrations in the UFM1 conjugation system that prevent dissociation of the 60S ribosomal subunit from the SEC61 translocon result in persistent binding of SEC61 to stalled ribosomes, which in turn causes prolonged occupancy of SEC61 channels by abnormal ribosome-nascent chain complexes [20,145,158]. This steric hindrance compromises the translocation and membrane integration of other nascent polypeptides, ultimately impairing secretory pathway function [20,159,160]. Therefore, the UFM1 conjugation system not only disrupts essential RQC quality control pathways but also leads to translocon dysfunction, failure of quality control mechanisms, and inefficient ribosome resource utilization, ultimately triggering cellular stress responses and pathological conditions resulting from RQC failure [20,145,161]. The resulting accumulation of aberrant ribosome–nascent chain complexes interferes with proteasomal degradation, causing protein aggregation that destabilizes the ER folding environment and triggers a cascade of ER stress, oxidative stress, and eventually caspase-dependent apoptosis [145,159,162].

However, a critical question remains unresolved: when cells simultaneously face “accumulation of mature misfolded proteins within the ER” and “ribosomal translation stalling”, how does the UFM1 conjugation system allocate its limited modification resources to address protein toxicity? Does it consistently prioritize ERAD, invariably favor RQC, or instead determine the processing priority based on the relative severity of each stressor? Addressing this question will provide a novel perspective for subsequent investigations into the underlying mechanisms by which the UFM1 conjugation system regulates PQC.

### 3.3. The UFM1 Conjugation System Activates Selective Cargo Packaging in the ER–Golgi Transport Process of Protein

Transport of proteins from the ER to the Golgi apparatus is a tightly regulated process essential for cellular homeostasis. Beyond the canonical coat protein complex II (COPII)-coated vesicles, accumulating evidence identifies the UFM1 conjugation system as an active regulator of cargo selection at ER exit sites, conferring specificity to ER–Golgi transport through cargo-adaptor recognition and spatially restricted UFMylation. This process is initiated with the recruitment of the Secretion-associated and Ras-related GTPase 1(Sar1) to the ER membrane [163,164]. Following its activation, Sar1 facilitates the assembly of COPII coat proteins, consisting of the Sec23/Sec24 complex and the Sec13/Sec31 complex, which mediate the coating of newly synthesized cargo proteins and vesicle budding [163]. GPCRs are cell surface receptors that recognize extracellular ligands [165]. Upon ligand binding, activated GPCRs initiate intracellular signaling cascades that regulate the recruitment and activation of the small GTPase Sar1 [165,166]. This Sar1-dependent process, in turn, controls COPII coat assembly and subsequent vesicle formation from the ER [164,166,167]. While this core machinery is sufficient for bulk protein secretion, selective export of certain cargos requires additional regulatory layers that determine which proteins are preferentially packaged into COPII vesicles. The UFM1 conjugation system provides such a layer by linking cargo identity to COPII engagement at ER. Alpha-2A adrenergic receptor (α_2_A-AR) is one type of GPCR that undergoes partial folding within the ER lumen. During this process, its cytosolic C-terminus (CT) binds directly to the UFBP1 cytosolic domain via hydrophobic and charge interactions, while the third intracellular loop (ICL3) specifically interacts with the WD40 domain of UFL1 [66]. The concurrent binding of UFL1 and UFBP1 results in the formation of a GPCR/UFBP1/UFL1 ternary complex [66,168]. Anchored to the ER membrane via its transmembrane domain, UFBP1 enriches this complex to ER exit sites (ERESs), through spatial proximity to Sec24A/B/D-coated COPII vesicles [66]. When GPCRs carry COPII recognition motifs, Sec24 directly recruits cargo by binding these motifs, followed by the polymerization of the Sec13-Sec31 outer coat, which drives membrane curvature and vesicle budding [18,43,66]. During this process, UFL1, functioning as an E3 ligase, catalyzes covalent conjugation of UFM1 to lysing 267 (K267) of UFBP1, enhancing GPCR loading efficiency by stabilizing complex conformation and improving spatial proximity to COPII machinery [43,66]. Post-transport, UFSP2 hydrolyzes the UFM1-UFBP1 linkage via its C302 cysteine protease activity, triggering complex disassembly in the Golgi apparatus and enabling UFBP1 recycling to the ER [18,60,66]. This cycle selectively sorts GPCRs harboring the UFBP1-binding α_2_A-AR, while GPCRs lacking this motif, such as Vesicular Stomatitis Virus Glycoprotein, utilize the default pathway mediated by Sec24 recognition of intrinsic export motifs [43,66]. These findings reveal a novel role of the UFM1 conjugation system in spatiotemporal membrane protein sorting. Vesicular transport is a core mechanism in eukaryotic cells, essential for fundamental cellular activities, particularly in maintaining cellular homeostasis and signal transduction [169]. This process relies on the UFM1 conjugation system for tagging and modifying cargo proteins, facilitating critical steps such as cargo enrichment, vesicle budding, and membrane fission [66]. The UFM1 conjugation system ensures precise coordination of GPCR biosynthetic trafficking with their functional maturation by selectively engaging conserved sorting signals, dynamically adapting vesicle formation to cellular demands, and synergizing with auxiliary proteins to maintain fidelity in secretion and receptor activity.

Cargos involved in ER–Golgi transport encompass a diverse range of types, including GPCRs, secretory proteins (such as insulin and amyloid precursor protein (APP)), and ion channels (such as ORAI1). Notably, the transport efficiency of different cargos is closely tied to cellular physiological needs. For example, pancreatic β-cells require efficient transport of insulin to respond to blood glucose fluctuations, while neurons demand precise sorting of APP to prevent aberrant cleavage and toxic Aβ fragment accumulation. Therefore, we hypothesize that the UFM1 conjugation system does not employ a “uniform regulatory mode” for all cargos. Instead, it achieves precise sorting through specific matching between unique cargo sorting codes and UFM1 modification sites. The core of this mechanism lies in the inherent differences in “sorting codes” across distinct cargos, and these differences directly determine the recruitment priority of the UFM1 conjugation system. This hypothesis not only elucidates the molecular basis for cargo-specific ER-Golgi transport but also provides novel insights for the future targeted delivery of specific drugs.

### 3.4. The UFM1 Conjugation System Promotes DNA Damage Repair by Amplifying ATM Activation Signals

DNA damage can arise from both endogenous factors, including reactive oxygen species (ROS) as metabolic byproducts and exogenous sources [170,171]. Recent studies have identified histone H4 UFMylation by UFM1 as a critical regulator of ataxia telangiectasia mutated (ATM) kinase activation [11,172]. Upon DNA double-strand breaks (DSBs), the MRE11/RAD50/NBS1 (MRN) complex recruits both ATM kinase and the UFM1 E3 ligase UFL1 to damage sites via the Forkhead-Associated/ BRCA1 C-Terminus domains of its Nijmegen breakage syndrome 1 protein (NBS1) subunit [11,173,174]. At DSB-proximal chromatin regions, UFL1 catalyzes mono-UFMylation of histone H4 at lysine 31, which is specifically recognized by the Hippo pathway kinase Serine/Threonine Kinase 38 (STK38) through its LIR/UFIM motif, leading to STK38-mediated recruitment of the SUV39H1-KAP1-HP1 complex that deposits local H3K^9^ trimethylation (H3K^9^me^3^) [11,173,174]. H3K^9^me^3^ subsequently binds the acetyltransferase Tip60 to acetylate ATM at lysine 3016 (ATM-K^3016^ac) for full kinase activation [11,173,174]. Concurrently, UFL1-mediated UFMylation of MRE11 at lysine 282 stabilizes the MRN complex conformation, facilitating ATM retention and sustained activation at DSBs [11,173,174]. Activated ATM phosphorylates UFL1 at serine 462 to enhance its E3 ligase activity, establishing a positive feedback loop that amplifies UFMylation signaling [11,173,174]. Ultimately, this feedback loop enables ATM to initiate cell cycle checkpoints, homologous recombination repair, and apoptotic pathways by phosphorylating checkpoint kinase 2 and histone H2A variant X. Moreover, H4 UFMylation may alter nucleosome stability or higher-order chromatin architecture to expose damaged sites and promote repair factor accumulation [11,173,174]. This reciprocal regulatory mechanism establishes a positive feedback loop that ensures rapid amplification of ATM activation signals, effectively coordinating DSB repair with cell cycle arrest to prevent genomic instability [11,175].

While existing studies have demonstrated that the UFM1 conjugation system is involved in DNA damage repair, many questions remain to be clarified. For instance, can the UFM1 conjugation system recognize different types of DNA damage (such as single-strand breaks/SSBs and UV-induced pyrimidine dimers)? Does its regulatory role in repair differ across different phases of the cell cycle? And what is the mechanism of its crosstalk with other DNA repair pathways (e.g., nucleotide excision repair/NER and base excision repair/BER)? All these questions provide new directions for future research.

### 3.5. The UFM1 Conjugation System Regulates Ferroptosis by Controlling the PIR Degradation Rate

Ferroptosis constitutes an iron-dependent regulated cell death pathway driven by the pathological accumulation of lipid peroxidation products and arises when antioxidant defenses fail to counterbalance excessive ROS production and polyunsaturated fatty acid oxidation. The UFM1 conjugation system has recently emerged as a critical upstream regulator of ferroptosis, primarily through the UFMylation-Pirin (PIR)-GPX4 axis. Under physiological or basal conditions, MCP enhanced UFL1-mediated UFMylation of PIR protein, specifically targeting the KKVT motif (K5/K6 sites) through a ubiquitinase cascade, thereby stabilizing PIR and preventing its proteasomal degradation [176,177,178,179]. Although the precise biochemical details of K5/K6-linked UFMylation remain unresolved, current evidence suggests that UFMylated PIR recruits nuclear factor I/C, binds the GPX4 promoter region (−2380 to −2241), and induces histone H3K27 acetylation to drive high GPX4 expression, thereby sustaining glutathione (GSH)-dependent lipid peroxide clearance, ultimately protecting cells from ferroptosis [178,179,180,181]. Moreover, loss of UFMylation sensitizes cells to ferroptosis indicates that UFL1 knockdown reduces PIR UFMylation, leading to proteasomal degradation of PIR and markedly reducing GPX4 transcription [178,179]. The resulting GPX4 deficiency causes GSH depletion and accumulation of Malondialdehyde and 4-Hydroxynonenal, and Fe^2+^ exacerbates polyunsaturated fatty acid peroxidation via the Fenton reaction, inducing ferroptosis-characteristic alterations such as mitochondrial membrane rupture and cristae disappearance [36,178,182]. Furthermore, UFL1 deficiency activates and induces nuclear receptor coactivator 4-dependent ferritinophagy, releasing free Fe^2+^ and amplifying lipid peroxidation, thereby establishing a ferroptosis–autophagy positive feedback loop [178,179]. Notably, in breast cancer, pathologically elevated MCP expression induces ferroptosis via an independent pathway by inhibiting UFMylation by the UFM1 conjugation system of Solute Carrier Family 7 Member 11, blocking cystine uptake and causing GSH synthesis failure [178,179]. This observation highlights substrate-specific differences in UFMylation by the UFM1 conjugation system across cancer types. Therefore, we suggest that future studies on the selective manipulation of specific UFMylation events may represent a promising anticancer strategy. Researchers could design “PIR UFMylation inhibitors” (such as antibodies that interfere with UFL1-MCP interaction) to selectively induce ferroptosis in cancer cells without affecting the protective UFM1-mediated regulation in normal cells.

### 3.6. UFM1 Conjugation System Control of Autophagic Flux and ER-Phagy

Autophagy is a highly conserved cellular degradation process, encompassing both general autophagy and selective autophagy such as ER-phagy [183,184,185]. Increasing data indicates that the UFM1 conjugation system functions as a key upstream regulator of the initiation phase of autophagy through UFMylation of autophagy–lysosome pathway targets, including valosin-containing protein (p97) [36,104,186]. Studies suggest that the UFM1 conjugation system regulates Beclin 1 (BECN1) through p97-dependent UFMylation [186]. BECN1, a core regulatory factor in the initiation phase of autophagy, serves as the central component of the class III phosphatidylinositol 3-kinase (PI3K) complex [187]. Beclin 1 recruits Phosphatidylinositol 3-kinase catalytic subunit type 3 (PIK3C3) and Autophagy-related protein 14 (ATG14) to catalyze the production of phosphatidylinositol-3-phosphate (PtdIns3P), a key lipid signal essential for autophagosome membrane nucleation and elongation [188,189]. UFMylation enhances BECN1 stability by modifying the K109 residue of p97, thereby activating the deubiquitinating enzyme ataxin-3 (ATXN3) [190,191]. Activated ATXN3 significantly reduces K48-linked ubiquitination of BECN1, thereby preventing its proteasomal degradation and elevating its intracellular protein levels [186]. Enhanced BECN1 stability promotes the efficient recruitment of PIK3C3 and ATG14, forming a functional PI3K complex that drives PtdIns3P synthesis and initiates autophagosome biogenesis [186,192]. Conversely, inhibition or dysfunction of the UFM1 conjugation system impairs UFMylation of p97, leading to insufficient activation of ATXN3 and resulting in excessive BECN1 degradation through the proteasomal pathways [186]. This cascade ultimately compromises autophagy flux by disrupting the critical UFM1-p97-ATXN3 regulatory axis required for BECN1 stabilization. Dysregulation of this pathway is implicated in neurodegenerative diseases and cancer. Pathogenic mutations in p97 that impair UFMylation compromise BECN1 stability and lead to autophagic defects, underscoring the clinical relevance of this regulatory mechanism [34,186,192].

As a specialized subtype of selective autophagy, ER-phagy operates with remarkable spatial precision to eliminate stressed or redundant ER subdomains while preserving functional ER networks, a regulatory feat orchestrated through UFM1-mediated substrate tagging and autophagosome recruitment [19,104]. Under diverse pathological stimuli including ER stress, nutrient deprivation, and oxidative damage, the ER membrane-localized enzyme Cytochrome b5 reductase 3 (CYB5R3) undergoes UFL1-UFBP1 complex-dependent UFMylation at lysine 214 (K214) [18,104]. This UFMylation modification disrupts the FAD-binding domain of CYB5R3, triggering a conformational transition from a closed (active) to an open (inactive) state [104]. The structural shift exposes the UFBP1-binding interface, enabling interaction with CDK5RAP3 through the UFM1-interacting motif (UFIM) of UFBP1 [104,125]. Subsequently, the LC3-interacting region motif of CDK5RAP3 recruits autophagosomal membrane-associated GABARAP/LC3 proteins, thereby directing the autophagosome to encapsulate the UFMylated CYB5R3-marked ER subdomains [104]. The autophagosome–lysosome fusion, which is dependent on core autophagy machinery including Autophagy-related 7, culminates in ER degradation [193,194]. When the UFM1 conjugation system is inhibited or defective, CYB5R3 cannot be UFMylated and degraded, resulting in ER-phagy failure and accumulation of misfolded proteins and damaged ER structures [104]. This accumulation exacerbates ER stress, disturbs proteostasis, and sustains abnormal redox activity on the ER membrane, collectively aggravating cellular injury and triggering apoptosis [195]. The pleiotropic roles of UFM1-dependent ER-phagy in stress adaptation, metabolic regulation, and disease progression underscore its centrality in cellular physiology, highlighting its potential as a therapeutic target for disorders of proteostasis and energy metabolism.

While existing studies have uncovered the core role of UFM1 in “autophagy-ER-phagy”, there remain critical scientific gaps. How does the member of the UFM1 conjugation system coordinate the dynamic balance between autophagic flux and ER-phagy in response to different stresses? Does it exert undiscovered regulatory effects during the autophagosome–lysosome fusion stage? And what is the mechanism of its crosstalk with other types of selective autophagy? Based on the synergistic characteristics of the autophagic network and the unresolved issues, the following reasonable hypotheses and prospects are proposed, providing a new perspective for mechanistic research and treatment of autophagy disorder-related diseases.

### 3.7. Distinct UFM1 Conjugation System Molecular Targets Define Its Opposite Roles in Acute Versus Chronic Inflammation

Inflammation constitutes an essential innate defense mechanism against pathogenic threats, and accumulating evidence indicates that the UFM1 conjugation system exerts context-dependent regulatory effects on inflammatory responses [196]. Notably, under chronic metabolic disorders, the UFM1 conjugation system promotes inflammation progression, whereas during acute inflammatory conditions, it suppresses inflammatory responses [75,197]. It is suggested that a molecular switch redirects UFM1 conjugation system regulatory output depending on inflammatory context. Current evidence suggests that the regulatory direction of UFM1 may depend on differential substrate engagement under distinct stress contexts. This context-dependent functionality highlights the complex regulatory mechanisms of UFM1 in inflammatory pathophysiology [75,198]. In lipopolysaccharide (LPS)-induced acute inflammation, Toll-like receptor 4 (TLR4) serves as the primary pattern recognition receptor, initiating NF-κB signaling upon activation [27]. Studies demonstrate that the UFM1 conjugation system suppresses LPS-triggered inflammatory responses by regulating the TLR4/NF-κB cascade [75,78]. UFM1 knockdown upregulated pyroptosis-related proteins and an effect reversed by TLR4-specific inhibitor TAK-242, confirming the TLR4-dependence of UFM1-mediated pyroptosis regulation in the inflammation response [27,199]. Conversely, UFM1 overexpression inhibits the LPS-activated TLR4-Myeloid differentiation primary response gene 88(MyD88)-Transforming growth factor beta-activated kinase 1 (TAK1) pathway, thereby suppressing pyroptosis and reducing the expression of NLRP3, caspase-1, and gasdermin D [27,75]. UFM1 may stabilize the interaction between NF-κB/IκBα by suppressing phosphorylation or ubiquitination-dependent degradation of IκBα, thereby preventing NF-κB nuclear translocation and reducing downstream cytokine expression (TNF-α, IL-6, VCAM-1) [124]. These effects are observed across diverse cell types, including endothelial cells, ovarian granulosa cells, and mammary epithelial cells, indicating a conserved mechanism of acute inflammatory suppression. Thus, in acute inflammation, UFM1 acts upstream of IκBα to directly restrain NF-κB activation.

**Figure 2:**
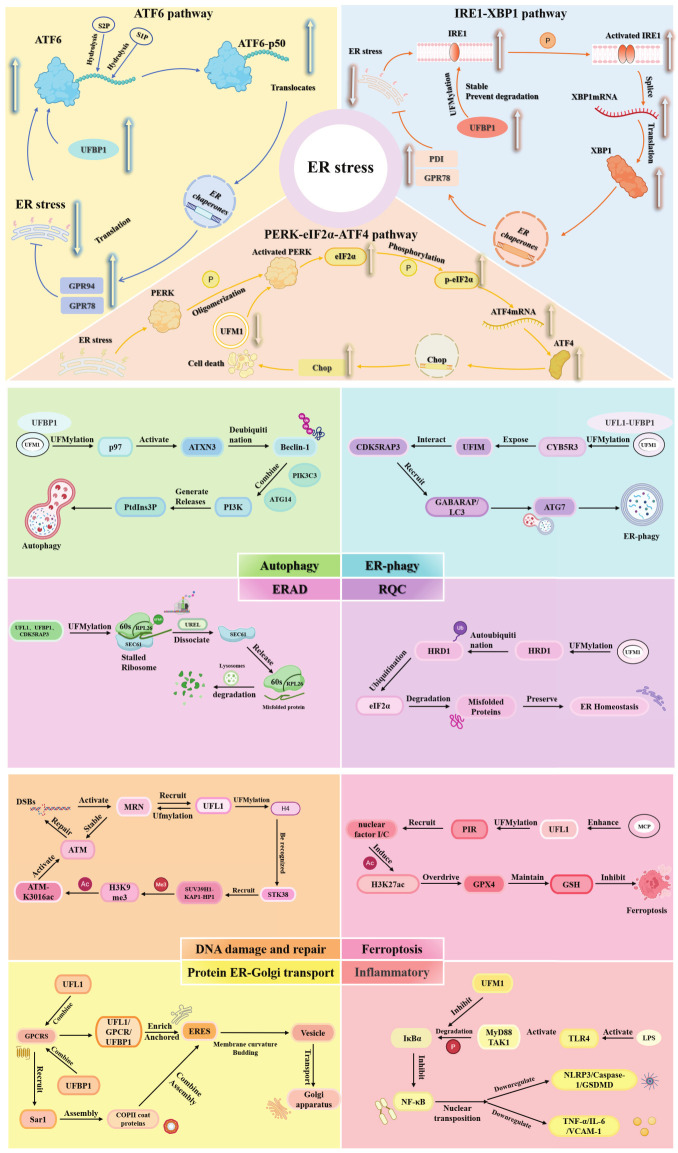
Schematic of regulatory cross-talk between UFM1 conjugation system and cellular pathways. UFM1 participates in diverse biological and physiological processes, including the ER stress response, modulation of autophagy and ER-phagy, involvement in protein quality control, participation in DNA damage repair, influence on ferroptosis progression, regulation of protein trafficking, and impact on the development of inflammatory responses.

In contrast, UFM1 plays a pro-inflammatory role in regulating inflammatory responses through distinct molecular mechanisms in chronic metabolic diseases such as diabetes [198]. This functional reversal arises from the engagement of CDK5RAP3, a known suppressor of NF-κB signaling [198,200]. Additionally, UFM1 expression upregulates via the JNK/ATF2-c-Jun pathway in response to hyperglycemia, creating a feed-forward loop that amplifies inflammation in diabetes. Co-immunoprecipitation assays in HEK293 and RAW264.7 cells confirmed the direct interaction between UFM1 and CDK5RAP3, indicating that elevated UFM1 expression directly facilitates CDK5RAP3 degradation via complex formation [198,200]. This reduction in CDK5RAP3 protein levels, without altering mRNA relieves its inhibition on NF-κB, leading to increased phosphorylation and transcriptional activity of the NF-κB subunit [198,200]. Concurrently, UFM1 enhances NF-κB signaling by promoting degradation of IκBα, thus promoting NF-κB nuclear translocation and the upregulation of TNF-α, IL-6, and IL-1β [198,200]. These mechanisms are validated through western blot analysis, luciferase reporter assays, and in vivo studies in *db/db* mice [198,200]. Collectively, UFM1 acts as a critical nexus linking metabolic stress to NF-κB-driven inflammation through dual regulation of CDK5RAP3 and IκBα [198,200]. However, how the UFM1 conjugation system precisely distinguishes the signal differences between “acute inflammatory responses” and “chronic inflammatory responses” and further switches its regulatory direction is still unknown. We hypothesize that the UFM1 conjugation system may determine its regulatory direction based on the expression levels of certain inflammatory factors or inflammasomes. This also provides a new perspective for future studies on the function of the UFM1 conjugation system in the inflammation response. For details about the role of members of the UFM1 conjugation system in various stress responses, see Figure 2.

## 4. The Regulation of UFM1 Conjugation System-Mediated UFMylation in Different Diseases and Therapeutic Implications

### 4.1. Neurological System

#### 4.1.1. Epileptic Encephalopathy

Epileptic encephalopathies (EIEEs) represent a heterogeneous group of epileptic syndromes characterized by refractory seizures, electroencephalographic abnormalities, and progressive neurological deterioration, often accompanied by cognitive, motor, and social dysfunction [50,201,202,203]. Mutations in UBA5 have emerged as a major cause of early onset of EIEE though the impairing of ER homeostasis, autophagy, mitochondrial integrity, and synaptic function. The mutations compound heterozygous c.1111G > A/p.Ala371Thr and c.562C > T/p.R188X of UBA5 impair thioester bond formation between UFM1 and UFC1, preventing UFM1 transfer to RPL26 [204,205,206]. RPL26 UFMylation deficiency compromises ER-associated quality control (ER-RQC), leading to activation of ER stress responses [204,205,206,207]. Persistent ER stress leads to hyperactivation of the IRE1α, resulting in excessive XBP1 mRNA splicing and accumulation of XBP1s, thereby enhancing neuroinflammation and seizure susceptibility [13,208,209,210,211]. XBP1s promotes neuroinflammation through NF-κB activation, which increases seizure susceptibility and accelerates neuronal dysfunction [13,208,209,210,212]. Concurrently, chronic ER stress recruits the IRE1α-TRAF2 complex driving JNK phosphorylation. It is a mechanistic bridge linking ER perturbation to mitochondrial apoptosis; phosphorylated JNK facilitates conformational activation of BAX, promotes its dissociation from BCL-2, and accelerates translocation of activated BAX to the mitochondrial outer membrane [133,204,206]. Consistent with this mechanism, UBA5 mutations disrupt BAX subcellular localization, facilitating its translocation to mitochondrial membranes and subsequent oligomerization into pores, triggering mitochondrial membrane potential (ΔΨm) collapse and hippocampal neuronal apoptosis [213,214,215,216,217]. The consequent opening of BAX pores results in the release of cytochrome c (CytC) and Smac/DIABLO into the cytosol, where CytC engages Apaf-1 to activate caspase-9 and caspase-3, culminating in irreversible neuronal apoptosis [214,215,216,217,218]. This integrated dysregulation of ER stress, mitochondrial apoptosis, and synaptic excitability underscores the multifaceted role of UFM1 conjugation system defects in epileptic encephalopathies.

#### 4.1.2. Alzheimer’s Disease

Alzheimer’s disease (AD) is a neurodegenerative disorder characterized by progressive degeneration of the central nervous system, with core pathological hallmarks including extracellular deposition of β-amyloid (Aβ) plaques and intracellular aggregation of hyperphosphorylated Tau protein, forming neurofibrillary tangles [219,220]. Emerging evidence indicates that the UFM1 conjugation system critically modulates AD pathogenesis by modifying proteins involved in proteostasis, DNA repair, and inflammation [96]. Studies have shown that elevated levels of UFM1 are observed in the temporal and frontal cortices of AD patients and strongly correlate with pathological Tau accumulation [35,96,206].

UFM1 modification of ribosomal protein RPL26 impairs proteasomal degradation of Tau, while UFMylation of the ERAD factor HRD1 (at Lys610) disrupts clearance of amyloid precursor protein and its toxic Aβ fragments [35,96]. Furthermore, UFMylation of the DNA repair protein MRE11 (at Lys282) by UFM1 reduces double-strand break repair efficiency, synergizing with pathological Tau’s sequestration of the repair factor 53BP1 to exacerbate genomic instability [35,96]. Together, these events favor intracellular accumulation of aggregation-prone proteins. Concurrently, UFMylated RPN1 and CYB5R3 disrupt ER-phagy, leading to a loss of autophagic clearance of Aβ/Tau and triggering CHOP-mediated apoptosis via misfolded protein accumulation [35,96,206]. Neuroinflammatory cascades are fueled by UFMylated PD-L1 and NF-κB activation, which induce microglial TLR4 hyperactivation, increased with a 3-fold increase in TNF-α/IL-1β levels, and block Aβ-clearing macrophage recruitment, while reduced levels of the UFMylation substrate 14-3-3ε in the AD temporal cortex intensify damage [35,96,206]. Thus, the UFM1 conjugation system directly targets Aβ/Tau-associated proteins and dysregulates DNA repair, autophagy, and immune pathways [206]. It can be hypothesized that specifically inhibiting UFM1-mediated modification of HRD1 at Lys610 (to reduce Aβ fragment accumulation) or enhancing UFMylation of 14-3-3ε (to alleviate Tau toxicity and neuroinflammation) could offer a new direction for precise intervention in Alzheimer’s disease.

### 4.2. Digestive System

#### 4.2.1. Hepatitis

Hepatitis is a common liver disorder arising from diverse factors, including viral infections, alcohol abuse, and drug toxicity [221]. Accumulating evidence indicates that multiple components of the UFM1 conjugation system, including UFM1, UBA5, UFC1, and UFBP1, are markedly downregulated in both alcoholic and non-alcoholic hepatitis [102,125,222]. The lower expression of UFM1 conjugation system members correlates positively with Mallory–Denk body accumulation, indicating that UFMylation deficiency exacerbates ER stress and disrupts protein quality control [125,222,223]. Site-specific UFMylation at K267 of UFBP1 is crucial for stabilizing IRE1α, thereby suppressing activation of the PERK-eIF2α-ATF4 and IRE1α-XBP1s-Caspase 2 cascades [125,222]. This attenuation mitigates ER stress-induced lipogenesis mediated by SREBP1/SCD1/DGAT2, insulin resistance, and hepatocyte damage, positioning the UFM1-UFBP1 axis as a key therapeutic target for orchestrating ER integrity and inflammatory balance in hepatitis [86,125,222]. UFBP1 loss not only intensifies UPR dysregulation and induces severe ER stress, activating the PERK-eIF2α-ATF4 and IRE1α-XBP1 inflammatory pathways to stimulate NF-κB-mediated release of IL-1β and TNF-α, recruit immune cells, and generate ROS, but also activates the mechanistic target of the rapamycin (mTOR) signaling pathway [86,125,222]. Elevated mTOR phosphorylation promotes downstream targets p70S6K and 4EBP-1, while concurrent disruption of the TGF-β/Smad3 pathway increases Smad3 phosphorylation, directly activating profibrotic genes like collagen [86,125,222]. These combined effects drive hepatic stellate cell (HSC) activation into myofibroblasts and excessive extracellular matrix collagen secretion. In a high-fat diet model, UFBP1 deficiency amplifies these pathologies, causing hepatic steatosis, inflammatory infiltration, and collagen deposition [86,125,222]. Importantly, clinical analyses suggest that reduced expression of UFL1/UFBP1 in human alcoholic hepatitis and cirrhosis tissues underscores their protective role against fibrosis development following hepatitis. We propose that targeted restoration of UFMylation at the K267 site of UFBP1 may simultaneously inhibit PERK-eIF2α-mediated ER stress and mTOR/Smad3-driven hepatic stellate cell activation, providing a novel direction for intervening in the progression of hepatitis to liver cirrhosis.

#### 4.2.2. Liver Cancer

Dysfunction of the UFM1 conjugation system directly promotes hepatocellular carcinoma (HCC) via sustained mTOR signaling activation [86,224,225]. Hepatocyte-specific UFL1 or UFBP1 knockout mice exhibited over 50% spontaneous HCC incidence at one and a half years old, characterized by liver nodules, foam cell accumulation, and elevated alpha-fetoprotein [86,224,226,227]. UFBP1/UFL1 deficiency disrupts their interaction with the mTOR/GβL complex, leading to constitutive mTOR phosphorylation and p-p70S6, p-4EBP-1, driving aberrant proliferation [86,224]. Consistently, in Diethylnirtosamine-induced hepatocellular carcinoma models (DEN), UFL1 loss markedly increased HCC susceptibility, coinciding with aberrant p-mTOR and higher expression of GβL [86,224]. Clinical analyses further confirmed that downregulated UFL1/UFBP1 expression in human HCC tissues inversely correlated with mTOR activation [86,224]. Notably, UFBP1 deficiency also promotes H3S10P phosphorylation, accelerating chromosome condensation and entry into the G2/M phase [86,224]. mTOR inhibitor rapamycin alleviates UFBP1 deficiency-induced liver injury and tumor progression, validating the UFM1-mTOR axis as a promising target for HCC therapy [86,224].

#### 4.2.3. Gastric Cancer

Gastric cancer, a malignant tumor originating from the gastric mucosal epithelium, maintains high global incidence and mortality rates, being particularly prevalent in East Asia [228]. Clinical analyses have revealed significantly downregulated transcript levels and protein expression of UFM1 in gastric cancer tissues compared to paired adjacent normal tissues and with higher UFM1 levels correlating with markedly improved 5-year survival outcomes [229]. UFM1 directly regulates with 3-phosphoinositide-dependent protein kinase 1 (PDK1), promoting its degradation via the ubiquitin–proteasome system (UPS) [107,228,229]. The resulting reduction in PDK1 protein levels suppresses phosphorylation of AKT at Thr308, a critical step for full PI3K/AKT pathway activation, while concurrently attenuating mTORC2-mediated phosphorylation at Ser473, leading to inhibition of AKT activity [100,107,229]. This inhibition of AKT further downregulates expression of Glycogen Synthase Kinase 3β, mTOR, and FOXO1, ultimately blocking epithelial–mesenchymal transition, invasion, migration, and survival of gastric cancer cells [230]. Co-downregulated expression of CDK5RAP3 and UFM1 exacerbates tumor malignancy through the synergistic suppression of AKT pathway phosphorylation and, when combined with TNM staging, significantly improves prognostic accuracy [107,229,231]. Importantly, the UFM1-CDK5RAP3-PDK1-AKT axis not only elucidates the molecular basis of gastric carcinogenesis but also offers potential biomarkers for patient stratification and prediction of platinum-based chemotherapy sensitivity [107,229,232]. These findings support the hypothesis that targeted upregulation of UFM1 or CDK5RAP3 expression in gastric cancer cells is expected to synergistically strengthen the inhibitory action of platinum-based chemotherapeutic drugs against the PI3K/AKT pathway while enhancing the precision of patient prognostic stratification, thus opening up a new intervention direction for the precise combination therapy of gastric cancer.

### 4.3. Reproductive System

#### 4.3.1. Breast Cancer

Breast cancer arises predominantly from epithelial cells of mammary ducts or lobules, and represents one of the most prevalent malignancies in women worldwide [233]. Accumulating evidence has revealed that the UFM1 conjugation system has tumor-suppressive roles reported in other malignancies. However, UFM1 acts as a pro-oncogenic modifier by stabilizing ERα and amplifying its transcriptional signaling. Lysine residues 171 and 180 (Lys171 and Lys180) of ERα have been identified as the primary sites modified by UFM1. UFM1 modification at these sites stabilizes ERα through two complementary and mechanistically distinct modes of competition with the ubiquitin. On the one hand, UFMylation directly occupies Lys171/180, thereby preventing their use as acceptor sites for K48-linked ubiquitin chains. This excludes E3 ligases from forming degradative ubiquitin modifications on ERα [234]. On the other hand, UFM1 forms poly-UFM1 chains via Lys69, generating a steric hindrance effect around the modification sites. This shields adjacent regions and alters local conformation, impeding access of the ubiquitination enzyme complex (E1/E2/E3) to catalytic interfaces. These cooperative mechanisms markedly reduce ERα ubiquitination, prolong its half-life, and enhance transcriptional activity, ultimately upregulating downstream targets such as pS2, cyclin D1, and c-Myc to promote tumor growth [99,235,236]. Clinically, UBA5, UFC1, UFL1, and UFBP1 are significantly upregulated in ERα-positive breast cancer cells and tissues, with expression levels positively correlated with histopathological malignancy grades, indicating that global upregulation of UBA5, UFC1, UFL1, and UFBP1 promotes breast cancer growth [99,237,238]. Experimental studies demonstrated that Silencing UBA5 or UFL1 decreases ERα UFMylation and accelerates its degradation via K48-linked ubiquitination [99,239]. In summary, these findings establish the UFM1 conjugation system as a pivotal regulator of ERα stability and a key driver of breast cancer progression, suggesting that targeting the UFM1–ERα axis may offer a promising therapeutic avenue for hormone-dependent breast cancer. These strategies are conceptually distinct from conventional endocrine therapies, as they modulate ERα protein homeostasis rather than ligand binding, offering a potential route to overcoming anti-estrogen resistance.

#### 4.3.2. Premature Ovarian Failure

UFL1 plays an important role in the maintenance of ovarian function by regulating endoplasmic reticulum (ER) calcium homeostasis in granulosa cells. In premature ovarian failure (POF), aberrant activation of the UFL1 disrupts intracellular calcium signaling primarily through differential regulation of stromal interaction molecules STIM1 and STIM2, two key sensors of ER calcium depletion [26,240,241,242]. When expression of UFL1 is aberrantly upregulated, the STIM1/calcium release-activated calcium channel (ORAI1) balance is disrupted, impairing store-operated calcium entry (SOCE)—the process that replenishes ER calcium after depletion [26,242,243]. Under healthy conditions, transmembrane and Coiled-Coil Domains 1 (TMCO1), a key calcium leak channel on the ER membrane, prevent ER calcium overload. However, overexpressed STIM1 or inhibited ORAI1 impairs TMCO1 function, leading to abnormal elevation of cytosolic calcium concentration though the calcium channel IP3 receptor [26,242,244]. Calcium overload directly triggers ER stress, activating the UPR and inducing pro-apoptotic factors CHOP and Caspase-12, ultimately leading to ovarian granulosa cell apoptosis via Caspase-9/3 activation. This process is closely associated with follicular atresia in premature ovarian failure [26,242,245]. Additionally, aberrant activation of UFL1 upregulates STIM2 expression in ovarian granulosa cells, which suppresses ORAI1-dependent SOCE calcium channels. Concurrently, persistent STIM2 activation triggers signaling pathways that suppress Transient Receptor Potential Canonical (TRPC) family function, further exacerbating ER calcium store depletion [26,242]. In compensation, cells increase cytosolic calcium influx via plasma membrane voltage-gated channels or mitochondrial calcium release, resulting in cytosolic calcium overload. This overload activates phospholipase A2, which hydrolyzes membrane phospholipids to generate arachidonic acid (AA), subsequently metabolized by cyclooxygenase and lipoxygenase to produce excessive reactive oxygen species (ROS) [26,242,245]. ROS not only directly impair the expression of GRP78 and PDI, impairing their folding capacity and aggravating the UPR, but also induce mitochondrial mPTP opening, releasing cytochrome C and activating the Caspase-9/3 cascade, driving irreversible apoptosis of ovarian granulosa cells and accelerating follicular atresia and ovarian functional decline [26,242].

### 4.4. Urinary System

#### 4.4.1. Kidney Injury

The UFM1 conjugation system plays a critical cytoprotective role in kidney injury by preserving ER proteostasis by mediated ER-phagy. Studies have shown that in different models of kidney injury, including contrast-induced, ischemia-reperfusion, cisplatin-induced, and folate-induced kidney injury, UFM1 is consistently downregulated, and its regulatory mechanism is consistently mediated by the UFM1-UFL1-UFBP1-dependent ER-phagy pathway [246,247,248]. Significantly reduced activity in acute kidney injury (AKI) leads to pathological accumulation of damaged ER, accompanied by upregulation of expression of GRP78 and CHOP [248,249]. Conversely, another study revealed that overexpression of UFBP1 or UFL1 restores ER-phagy flux, alleviates ER stress and apoptosis, and improves cellular viability in HK-2 renal tubular epithelial cells [102,248]. UFBP1 recruits UFL1 to ER membranes to initiate UFMylation, thereby activating ER-phagy for selective clearance of dysfunctional ER and ultimately mitigating tubular epithelial cell injury [248]. Notably, renal tubule-specific *Ufl1* knockout mouse models have further revealed that UFL1 deficiency induces renal atrophy, interstitial fibrosis, and impaired renal function, accompanied by activation of the PERK-eIF2α signaling pathway, upregulation of BAX, and downregulation of Bcl-2 [248,250]. Furthermore, consistent with in vivo results, UFL1 knockdown in HK-2 cells similarly triggered PERK-eIF2α pathway activation and elevated expression of apoptotic markers [250]. In summary, these findings demonstrate that the UFM1 conjugation system preserves renal homeostasis by maintaining ER proteostasis, facilitating UFMylation-dependent ER-phagy, and suppressing stress-induced apoptosis. This highlights UFMylation as a critical cytoprotective mechanism in kidney injury.

#### 4.4.2. Renal Cell Carcinoma

Renal cell carcinoma (RCC), characterized by high malignancy and poor prognosis, is closely linked to dysregulation of the tumor suppressor Phosphoprotein 53 (p53) [251]. A study on the Mouse Double Minute 2 Homolog (MDM2), the E3 ubiquitin ligase of p53, suggested that MDM2 and p53 co-interact and are pivotal regulators in the pathogenesis and progression of RCC [252,253]. Research revealed that UFMylation competitively regulates UFL1-DDRGK1 complex bindings to the N-terminal domain of p53, sterically hindering MDM2 from interacting with p53 [254]. Moreover, UFMylation modifies four specific C-terminal lysine residues, including Lys351, Lys 357, Lys 370, and Lys 373 of p53, which are also targeted by MDM2 for ubiquitination [254]. By occupying these sites, UFMylation directly antagonizes MDM2-mediated ubiquitination and subsequent proteasomal degradation of p53 [22,254]. This stabilization enhances p53’s transcriptional activity, suppressing tumor cell proliferation and in vivo tumorigenesis. Clinical cohort analyses have confirmed a significant downregulation of UFL1 and DDRGK1 in RCC tissues, which correlates with reduced p53 protein levels [22,34,254]. This finding suggests that impaired UFMylation disrupts p53 homeostasis, driving RCC progression [254].The competitive interplay between UFMylation and ubiquitination unveils a promising therapeutic axis for targeting RCC via the UFL1-DDRGK1-p53 pathway [254].

### 4.5. Cardiovascular System

#### 4.5.1. Heart Disease

Cardiac disorders represent a major global health burden, often associated with ER stress and maladaptive remodeling [255,256,257]. Studies have suggested that the UFM1 conjugation system may attenuate pathological cardiac progression by maintaining maintenance of ER proteostasis, suppression of fibrotic remodeling, and coordination of autophagic clearance pathways [256,258,259]. The UFM1 conjugation system dynamically responds to myocardial ischemia, with its expression level critically determining cardiac outcomes. In ischemia–reperfusion models, UFL1 expression is significantly downregulated in cardiomyocytes particularly within necrotic zones, which correlates with impaired cell survival and exacerbated cardiac damage [7,256]. This is a pattern similarly observed in human failing hearts from dilated cardiomyopathy patients. UFL1 deficiency activates ER stress responses by impairing PERK-eIF2α-ATF4 signaling, leading to early cardiac dysfunction. Conversely, compensatory UFL1 upregulation suppresses atrial natriuretic peptide and brain natriuretic peptide while mitigating maladaptive cardiac growth under pressure overload conditions. In contrast, UFL1 knockout mice develop progressive ventricular dilation interstitial fibrosis and fetal gene reactivation [7,256]. Cardiac-specific Ufl1 knockout mice exhibit age-dependent interstitial fibrosis, accompanied by elevated levels of GRP78 and CHOP) which activates TGF-β/Smad3 pathway signaling to promote collagen deposition [7,256,260]. Necrotic signaling further amplifies fibrosis, while transcriptomic analyses reveal dysregulation of ER-resident genes involved in protein folding and redox homeostasis, linking unresolved UPR activation to fibrogenesis [7,256]. These findings suggest that the UFM1 conjugation system plays a pivotal role in modulating ER stress and conferring cardioprotection; it emerges as a promising novel therapeutic target for the management of heart diseases. By modulating the activity of the UFM1 conjugation system, it may be possible to reduce ER stress levels in patients with heart diseases and enhance cardiac function.

#### 4.5.2. Diseases of Erythroid Differentiation and Development

Disruption of hematopoietic differentiation leads to lineage-specific developmental arrest, with the members of the UFM1 conjugation system playing a crucial role in the differentiation and development of blood cells [45,261,262]. Current research has demonstrated that the loss of UBA5, UFBP1, or UFL1 results in abnormal development and differentiation of blood cells, albeit at different stages and via distinct mechanisms [263,264,265]. UBA5 deficiency impairs differentiation of common myeloid progenitors (CMPs) into megakaryocyte–erythroid progenitors (MEPs), leading to severe embryonic anemia characterized by reduced erythrocytes, multinucleated erythroblasts, and decreased megakaryocytes [263,265]. Furthermore, UBA5 deficiency impairs the expression of genes related to erythroid transcription, including the master erythroid regulator GATA binding protein 1 (GATA1), Friend of GATA1, and Erythropoietin Receptor, thereby inhibiting the viability of erythroid cells [265]. UBA5 loss also induces aberrant global UFMylation, triggering eIF2α phosphorylation and GRP78 upregulation, which in turn triggers BAX and PUMA to mediate apoptosis, causing massive loss of erythroid progenitors and reduced erythropoiesis [265]. Additionally, UBA5 deficiency indirectly affects the UFMylation modification of ASC1, preventing ASC1 from effectively binding to the GATA1 and Krüppel-like Factor 1 promoters, further suppressing erythroid gene expression. Although the mechanisms by which UFBP1 deficiency causes abnormal hematopoietic cell differentiation and development to partially overlap with those of UBA5 deficiency, UFBP1 deficiency primarily blocks the differentiation of colony-forming unit–erythroid (CFU-E) into proerythroblasts and does not significantly affect the MEP compartment [264,265]. Furthermore, since UFBP1 acts both as a cofactor for UFL1 and as a UFMylation target, it directly mediates the UFMylation modification of ASC1, thereby influencing ASC1’s nuclear localization and subsequent binding to GATA1 and KLF1 [263,264]. Moreover, unlike UBA5 or UFBP1 deficiency, which primarily affects erythroid/megakaryocytic differentiation and development, UFL1 deficiency causes comprehensive impairment of the differentiation of HSCs into progenitors, leading to HSC exhaustion, DNA damage accumulation, p53 activation, and mitochondrial dysfunction [263,264]. This significantly differs from the effects of UBA5 or UFBP1 deficiency, where HSC apoptosis is primarily driven by ER stress without DNA damage or autophagy defects [263]. Similar to UBA5 deficiency, UFL1 deficiency indirectly affects ASC1 function through UFMylation modification, rather than directly influencing ASC1 to affect erythroid-related gene expression and transcription as UFBP1 does [263]. Crucially, UFL1 is the only component among the three that influences hematopoietic cell differentiation and development by regulating autophagy and DNA damage repair [263]. These results hypothesize that targeted activation of UFL1-mediated UFMylation at the K136 site of ribosomal protein RPL26 may simultaneously alleviate the accumulation of DNA damage in hematopoietic stem cells (HSCs) and the differentiation arrest of erythroid precursors caused by UFL1 deficiency. This modification may not only reduce stress-induced damage in HSCs by improving ribosome quality control but also restore the differentiation of CFU-E into proerythroblasts, providing a new intervention strategy for hereditary erythropoietic disorders.

### 4.6. Skeletal System

**Figure 3:**
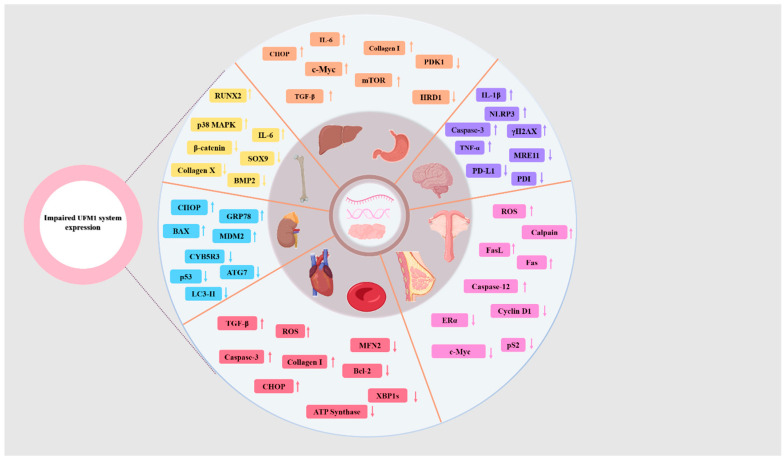
Schematic diagram of UMF1 conjugation system in the pathogenesis of various diseases. The UFM1 conjugation system modulates pathogenesis across various organ systems by regulating key factors.

Spondyloepimetaphyseal dysplasia, type Di Rocco, is characterized by disproportionate short stature, genu varum, gait instability, lumbar hyperlordosis, and coxa vara. Radiological features include epiphyseal and metaphyseal dysplasia, often necessitating hip joint replacement [266]. Cys302Ser mutations in UFSP2 have been identified as the genetic cause of Spondyloepimetaphyseal dysplasia, type Di Rocco. This mutation abolishes UFSP2 protease activity, thereby preventing cleavage of pro-UFM1 into its mature form and blocking exposure of the conserved C-terminal glycine (Gly83) essential for conjugation [267]. Consequently, loss of mature UFM1 consequently disrupts UFMylation-dependent protein modification, leading to dysregulated ER stress response, defective collagen trafficking, and impaired chondrocyte differentiation, which collectively contribute to vertebral deformities and defective long bone mineralization in Spondyloepimetaphyseal dysplasia, type Di Rocco [133,267]. These findings underscore that precise UFMylation regulation is essential for skeletal morphogenesis and homeostasis. Beyond developmental skeletal disorders, data suggest that UFL1 plays an important role in osteoarthritis (OA) development [268]. UFL1 expression is significantly reduced in OA cartilage, and this triggers nuclear translocation of FOXO1 and activates the cell cycle inhibitor p21 (CDKN1A), resulting in chondrocyte senescence [268]. Moreover, UFL1 deficiency also induces phosphorylated NF-κB activation, which enhances the expression of senescence-associated secretory phenotype (SASP) factors, including MMP13, IL-6, and ADAMTS4, thereby accelerating extracellular matrix degradation (Figure 3) [124,268,269]. Panaxatriol, a natural compound, binds UFL1 at Ser312, stabilizing UFL1 and blocking its degradation, reducing P21 and SASP expression to mitigate senescence and matrix catabolism [87,268,270]. Future studies should explore the potential role of the UFM1 conjugation system in bone remodeling, mineral metabolism, and fracture repair to uncover novel therapeutic strategies targeting UFMylation in skeletal disorders.

## 5. Conclusions

It has been found that number of UFM1 conjugation systems are highly conserved among species and are widely expressed in mammals [57,158]. The UFM1 conjugation system, which involves UFM1, UBA5, UFC1, UFL1, deconjugating proteases UFSP1/2, and CDK5RAP3, ensures precise spatiotemporal control of UFMylation; this system enables precise spatiotemporal control of UFMylation, thereby dynamically regulating substrate stability, localization, and signaling competence. Mounting evidence indicates that the UFM1 conjugation system is a pivotal regulatory network governing all kinds of stress responses to mediate disease pathogenesis, including inflammation and tumorigenesis. Given its central role in coordinating proteostasis and signaling fidelity, the UFM1 conjugation system holds considerable promise as a therapeutic target in translational medicine. Future research should focus on elucidating substrate specificity and structural determinants of UFM1 target recognition, developing isoform-selective UFMylation modulators to finely tune system activity, and exploring combinatorial therapeutic strategies that harness UFMylation’s cytoprotective effects while mitigating its pathogenic activation.

## Figures and Tables

**Table 1:** Structure and function of the UFM1 conjugation system.

Protein Name	Alternative Name	Molecular Weight (kDa)	Key Structural Features	Subcellular Localization	Main Effects	References
UFM1	Without	9.9	β-grasp fold C-terminal extension region	Cytoplasm Nucleus Cytosol	Maintenance of ER homeostasis Regulation of DNA damage response and repair mechanisms Modulation of inflammatory responses and immune regulatory pathways Embryonic development and nervous system functional regulation Tumorigenesis initiation and metastatic progression Autophagic flux and proteasome-mediated protein degradation	[5,7,17,21,27,41,44,54,67,70,71,72,73,74,75,76]
UBA5	UBE1DC1	46.2	Adenylation Domain N-terminal Extension homodimeric structure UFM1-interacting sequence C-terminal functional domain	Cytoplasm Nucleus Golgi apparatus Cytosol	Activation and transfer of UFM1 Regulation of the stability and efficiency of the UFM1 modification cascade Mutations disrupting the UFMylation process contribute to the pathogenesis of certain disorders	[16,41,42,44,67,73,77,78,79,80,81,82]
UFC1	HSPC155	19.4	N-terminal helical domain UBA5 binding interface Catalytic core domain	Extracellular exosome	The intermediary carrier role of UFM1 Coordination of E1-E3 cascade reactions Maintenance of homeostasis in the UFMylation reaction Involvement in ER stress and cellular homeostasis regulation	[5,15,16,40,43,50,51,83,84]
UFL1	RCAD Maxer NLBP KIAA0776	90	WH domain N-terminal helix	Cytosol Endomembrane system Endoplasmic reticulum membrane	Maintenance of ER homeostasis and function; Cardioprotective effects Regulation of inflammation and immune responses Support for neuronal survival and nervous system functionality	[4,16,18,43,55,71,85,86,87,88,89,90,91]
UFSP1	Without	23	Catalytic protease domain	Cytosol	Maturation of pro-UFM Regulation of UFMylation system activation	[6,14,67,92,93]
UFSP2	Without	46	Catalytic Domain N-terminal Domain	Nucleus Cytoplasm Endoplasmic reticulum	Mediation of UFM1 precursor maturation Execution of deUFMylation reactions Regulation of UFMylation signaling pathway homeostasis Direct association with the pathological mechanisms of certain disorders, such as neurological and skeletal developmental diseases	[6,14,62,67,94,95,96,97,98,99]
UFBP1	DDRGK1 C20 f11676 Dashurin	35.6	Proteasome COP9 eIF3 WH	Endoplasmic reticulum Endoplasmic reticulum membrane Nucleolus	Functioning as the core adapter protein of the UFM1 E3 ligase complex Modulation of ER stress and protein homeostasis Maintenance of multi-tissue and organ homeostasis Regulation of protein trafficking and secretion Involvement in disease-related pathological processes, such as cancer and neurological disorders	[5,8,16,32,33,55,76,77,97,100,101,102,103,104,105,106,107,108]
CDK5RAP3	C53 LZAP	53	C-terminal UUBD RBD that interacts with RPL26 Additional interface mediating UFL1/UFBP1 complex engagement	Protein-containing complex Endoplasmic reticulum membrane Centrosome Nucleus Cytoplasm Microtubule Cytosol Nucleolus	Functioning as a substrate adaptor of the UFMylation system Maintenance of ER homeostasis and stress responses Regulation of the cell cycle, metabolism, and cancer progression Acting as a core component of the E3 ligase complex Involvement in embryonic development and nervous system functionality	[8,13,18,19,20,68,69,104,105,106,107,108,109,110,111,112,113,114,115,116,117,118,119,120]

## Data Availability

The conclusions presented in this review are based on comprehensive literature synthesis and critical analysis, with no original datasets being collected or analyzed during the preparation of this work.

## Abbreviations

AA	Arachidonic acid
AD	Alzheimer’s disease
ASC1	Activating signal cointegrator 1
ATG14	Autophagy-related protein 14
ATM	Ataxia telangiectasia mutated
ATXN3	Ataxin-3
Aβ	β-amyloid
BAX	BCL2-associated X protein
BECN1	Beclin 1
BMP2	Bone Morphogenetic Protein 2
BmUFBP1	Bombyx mori UFBP1
CDK5RAP3	CDK5 regulatory subunit associated protein 3
CHOP	C/EBP Homologous Protein
COPII	Coat protein complex II
CT	C-terminus
CytC	Cytochrome c
CYB5R3	Cytochrome b5 reductase 3
DSBs	DNA double-strand breaks
ΔΨm	Mitochondrial membrane potential
E3	Ubiquitin ligases
EIEE	Epileptic encephalopathies
ER	Endoplasmic Reticulum
ERAD	Endoplasmic reticulum-associated degradation
GATA1	GATA binding protein 1
GPCRs	G protein-coupled receptors
GRP78	Glucose-Related Protein 78
GRP94	Glucose-Related Protein 94
GSH	Glutathione
GPX4	Glutathione peroxidase 4
H3K^9^me^3^	H3K^9^ trimethylation
H4	Histone 4
HCC	Hepatocellular carcinoma
HECT	Homologous to E6AP C-Terminus
HRD1	HMG-CoA reductase degradation protein 1
HSC	Hepatic stellate cell
ICL3	Third intracellular loop
MCP	Macrophage-capping protein
MDM2	Mouse Double Minute 2 Homolog
MEP	Megakaryocyte-erythroid progenitors
MRN	MRE11/RAD50/NBS1
mTOR	Mechanistic target of rapamycin
MyD88	Myeloid differentiation primary response gene 88
NBS1	Nijmegen breakage syndrome 1 protein
OA	Osteoarthritis
ORAI1	Calcium release-activated calcium channel
P21	Cyclin-dependent kinase inhibitor 1A
p53	Phosphoprotein 53
p97	Valosin-containing protein
PDI	Protein Disulfide Isomerase
PDK1	3-phosphoinositide-dependent protein kinase 1
PI3K	Phosphatidylinositol 3-kinase
PIK3C3	Phosphatidylinositol 3-kinase catalytic subunit type 3
PIR	Pirin
PQC	Protein quality control
PTM	Post-translational modification
PtdIns3P	Phosphatidylinositol-3-phosphate
RBD	Ribosomal association domain
RCC	Renal cell carcinoma
RING	Really Interesting New Gene
ROS	Reactive oxygen species
RPL26	Recombinant human ribosomal protein L26
RQC	Ribosome-associated quality control
S1P	Site-1 protease
S2P	Site-2 protease
Sar1	Ras-related GTPase 1
SASP	Senescence-associated secretory phenotype
SEC61	Sec61 complex
SOCE	Store-operated calcium entry
Sox9	SRY-related HMG Box 9
STAT3	Signal transducer and activator of transcription 3
STIM	Stromal interaction molecule
STK38	Serine/Threonine Kinase 38
TLR4	Toll-like Receptor 4
TMCO1	Transmembrane and Coiled-Coil Domains 1
TRPC	Transient Receptor Potential Canonical
UB	Ubiquitin
UBLs	Ubiquitin-like modifications
UBA5	Ubiquitin-like protein activating enzyme 5
UFC1	UFM1-conjugating enzyme 1
UFD	Ubiquitin-fold domain
UFIM	UFM1-interacting motif
UFM1	Ubiquitin Fold Modifier 1
UFL1	UFM1-specific ligase 1
UFSP1	UFM1-specific protease 1
UFSP2	UFM1-specific protease 2
UPS	Ubiquitin-proteasome system
UPR	Unfolded Protein Response
UUBD	UFM1/UFBP1-binding domain
WH	Winged helix
α_2_A-AR	Alpha-2A adrenergic receptor
